# The Effect of Gene Editing by CRISPR-Cas9 of miR-21 and the Indirect Target MMP9 in Metastatic Prostate Cancer

**DOI:** 10.3390/ijms241914847

**Published:** 2023-10-03

**Authors:** Juliana A. Camargo, Nayara I. Viana, Ruan Pimenta, Vanessa R. Guimarães, Gabriel A. dos Santos, Patrícia Candido, Vitória Ghazarian, Poliana Romão, Iran A. Silva, Alexander Birbrair, Miguel Srougi, William C. Nahas, Kátia R. Leite, Ericka B. Trarbach, Sabrina T. Reis

**Affiliations:** 1 Laboratory of Medical Investigation (LIM 55), Urology Department, Medicine School, University of Sao Paulo (FMUSP), São Paulo 01246-903, SP, Brazil; nayara_viana_2@hotmail.com (N.I.V.); ruanpimenta22@gmail.com (R.P.); vanessarguima@hotmail.com (V.R.G.); arantes_gabriel@hotmail.com (G.A.d.S.); patriciacandido11@gmail.com (P.C.); vitoriaghz@gmail.com (V.G.); romaosilva.poliana@gmail.com (P.R.); iransilva@gmail.com (I.A.S.); srougi@srougi.com.br (M.S.); katiaramos@usp.br (K.R.L.); sabrinareis@usp.br (S.T.R.); 2 Department of Bioscience, Minas Gerais State University (UEMG), Passos 37900-106, MG, Brazil; 3 D’Or Institute for Research and Education (ID’Or), São Paulo 04501-000, SP, Brazil; 4 Department of Pathology, Federal University of Minas Gerais, Belo Horizonte 30190-002, MG, Brazil; birbrair@wisc.edu; 5 Department of Dermatology, University of Wisconsin-Madison, Madison, WI 53715, USA; 6 Department of Radiology, Columbia University Medical Center, New York, NY 10032, USA; 7 Uro-Oncology Group, Urology Department, University of São Paulo Medical School and Institute of Cancer Estate of São Paulo (ICESP), São Paulo 01246-000, SP, Brazil; wnahas@uol.com.br; 8 Laboratory of Cellular and Molecular Endocrinology (LIM25), Endocrinology Departament, Medicine School, University of São Paulo (FMUSP), São Paulo 01246-903, SP, Brazil; ericka.trarbach@hc.fm.usp.br

**Keywords:** CRISPR-Cas9, miR-21, matrix metalloproteinases, metastatic prostate cancer

## Abstract

Prostate cancer (PCa) has a high prevalence and represents an important health problem, with an increased risk of metastasis. With the advance of CRISPR-Cas9 genome editing, new possibilities have been created for investigating PCa. The technique is effective in knockout oncogenes, reducing tumor resistance. MMP9 and miR-21 target genes are associated with PCa progression; therefore, we evaluated the MMP-9 and miR-21 targets in PCa using the CRISPR-Cas9 system. Single guide RNAs (sgRNAs) of MMP9 and miR-21 sequences were inserted into a PX-330 plasmid, and transfected in DU145 and PC-3 PCa cell lines. MMP9 and RECK expression was assessed by qPCR, WB, and IF. The miR-21 targets, integrins, BAX and mTOR, were evaluated by qPCR. Flow cytometry was performed with Annexin5, 7-AAD and Ki67 markers. Invasion assays were performed with Matrigel. The miR-21 CRISPR-Cas9-edited cells upregulated RECK, MARCKS, BTG2, and PDCD4. CDH1, ITGB3 and ITGB1 were increased in MMP9 and miR-21 CRISPR-Cas9-edited cells. Increased BAX and decreased mTOR were observed in MMP9 and miR-21 CRISPR-Cas9-edited cells. Reduced cell proliferation, increased apoptosis and low invasion in MMP9 and miR-21 edited cells was observed, compared to Scramble. CRISPR-Cas9-edited cells of miR-21 and MMP9 attenuate cell proliferation, invasion and stimulate apoptosis, impeding PCa evolution.

## 1. Introduction

Prostate cancer (PCa) is one of the most common cancers among men, accounting for 26% of all cases diagnosed and 11% of estimated deaths worldwide. Metastatic PCa is challenging to treat because it presents considerable heterogeneity and limited therapeutic response [1]. Presently, standard PCa treatment is based on androgen receptor (AR) deprivation. However, this is ineffective when the disease advances to the metastatic stage due to androgen resistance [2].

The ECM-degrading enzyme metalloproteinase 9 (MMP9) plays an important role in promoting the invasion of tumor cells into other tissues; thus, it directly contributes to carcinogenesis. It has been proposed that altered MMP9 expression may contribute to PCa evolution and, consequently, high metastatic potential [3]. In addition to the extracellular matrix, MMP9 also regulates integrins, transmembrane proteins involved in adhesion to the extracellular matrix, such as CDH1, ITGB1 and ITGB3 [4]. It has also been shown that RECK negatively regulates MMP9. Notably, studies have demonstrated that MMP9 gene expression could be regulated by a microRNA (miRNA) that is upregulated in PCa, resulting in RECK expression downregulation and MMP9 upregulation [5,6,7].

miRNAs are 18–22 nucleotide stretches of noncoding RNA that regulate posttranscriptional gene expression. One miRNA can silence multiple genes and is largely tissue-specific. Concerning cancer, miRNAs can act as a tumor suppressor when they function to silence oncogenes [8].

Furthermore, miR-21 is highly expressed in PCa, which could attenuate tumor suppressor gene expression [9,10]. Studies that definitively block this miRNA and evaluate tumor evolution have not been conducted. This miR-21 regulates the expression of multiple tumor suppression-related genes, such as RECK, which regulates MMP9. miR-21 also regulates PDCD4 and BTG2, which are apoptosis-related, and MARCKS, which controls cellular invasion [11,12,13]. 

A new gene-editing tool named clustered regularly interspaced short palindromic repeat (CRISPR)—associated Cas9 (CRISPR/Cas9) is a revolutionary genome-editing technology that can modify the genome of cells with high specificity, correcting mutations or deleting oncogenes [14]. In this sense, employing the CRISPR-Cas9 system to inactivate metastasis-related genes to promote cell survival could represent a novel strategy for improving treatment efficiency. However, the effect of blocking MMP9 and miR-21 in PCa for therapeutic purposes is still controversial. Bodey B et al. and Babichenko II et al. did not observe any difference in MMP9 expression in prostate cancer patients compared to the control group [15,16]. Similarly, Folini M. et al., also found no difference in the expression of miR-21 in prostate cancer [17]. Therefore, in the present study, we sought to investigate the effect of downregulating the expression of these markers using the CRISPR-Cas9 system in a metastatic PCa model.

## 2. Results

### Gene Editing with CRISPR/Cas9

**Figure 1:**
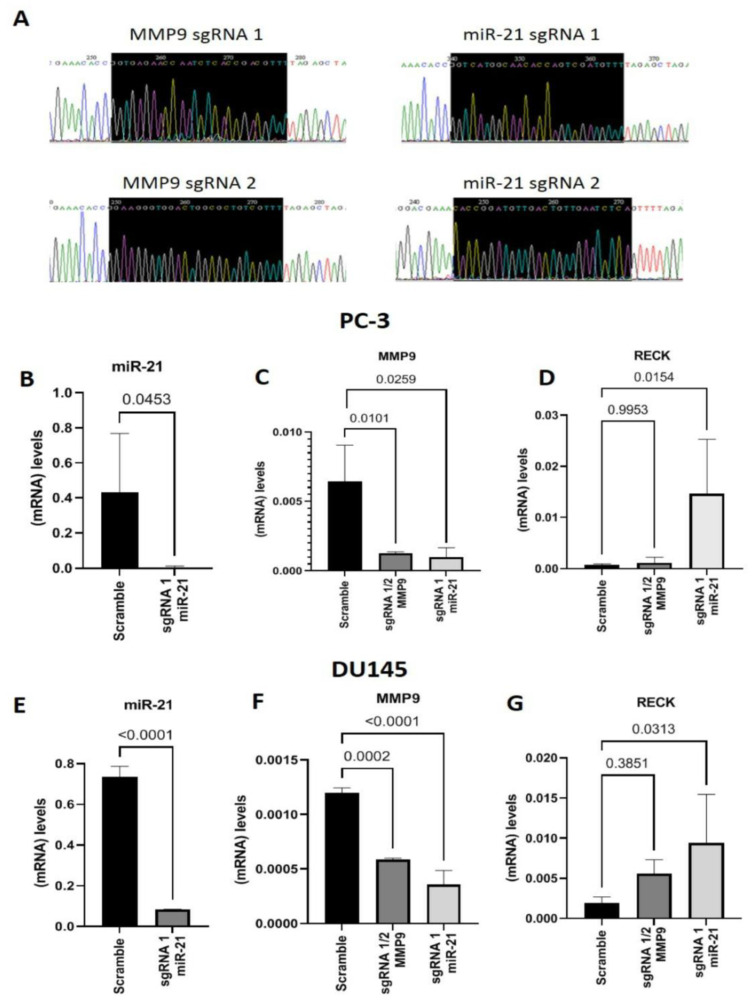
MMP9 and miR-21 gene editing with CRISPR-Cas9. (**A**) DNA sequencing of the PX-330 plasmid with the sequence inserts for MMP9 (sgRNA1 and sgRNA2) at the beginning of MMP9 Exon 1 on chromosome 20q (**left**) and DNA sequencing of the sequence inserts for miR-21 (sgRNA 1 and sgRNA2), located in two regions of chromosome 17 (**right**). (**B**) miR-21 gene expression in samples edited with miR-21 sgRNA 1 and their respective control transfected with the plasmid without any insert (Scramble) in the PC-3 cell line. (**C**,**D**) MMP9 and RECK gene expression in samples edited with CRISPR-Cas9 MMP9, sgRNAs 1 and 2 or miR-21 sgRNA1 compared with the scramble control in the PC-3 cell line. (**E**) miR-21 gene expression in samples edited with CRISPR-Cas9 miR-21 sgRNA 1 compared to the scramble control in the DU145 cell line. (**F**,**G**) MMP9 and RECK gene expression in samples edited with CRISPR-Cas9 MMP9 sgRNAs 1 and 2 or miR-21 sgRNA1 compared to Scramble in the DU145 cell line. Statistical significance set at *p* < 0.05.

We inserted sgRNAs into the PX-330 plasmid and sequenced them to validate the construct (Figure 1A). Before transfecting the plasmids into PC-3 and DU145 cell lines, we performed a puromycin dose–response curve and observed that 150 µg/mL for 10 days was the ideal concentration and time for selecting plasmid-transfected cells (Supplementary Figure S2). Cells were co-transfected with the plasmids containing MMP9 sgRNAs 1 and 2, targeting MMP9 Exon 1, or miR21 sgRNA 1 or 2. The transfections were validated with GFP images (Supplementary Figure S3).

As shown in Figure 1B, PC-3 cells transfected with miR-21 sgRNA 1 displayed downregulated miR-21 expression compared to the control group transfected with the plasmid without sgRNAs (Scramble) (*p* = 0.0453). MMP9 expression was significantly reduced in PC-3 cells co-transfected with MMP9 sgRNAs 1 and 2 or miR-21 sgRNA 1 compared to Scramble (*p* = 0.0101 and *p* = 0.0259, respectively) (Figure 1C). RECK expression remained unchanged in cells co-transfected with MMP9 sgRNAs 1 and 2. In contrast, RECK expression was significantly upregulated in cells transfected with miR-21 sgRNA 1 compared to Scramble (*p* = 0.0154) (Figure 1D). 

Similarly, in the DU145 cell line, miR-21 expression was downregulated in cells with CRISPR-Cas9-edited miR-21 compared to Scramble (*p* < 0.0001) (Figure 1E). MMP9 gene expression was downregulated in cells with CRISPR-Cas9-edited MMP9 (*p* = 0.0002) and miR-21 (*p* < 0.0001) compared to Scramble (Figure 1F). Although RECK expression was increased in CRISPR-Cas9 MMP9 edited cells, this change failed to reach a level of significance. In contrast, miR-21 CRISPR-Cas9-edited cells displayed significantly higher RECK expression levels (*p* = 0.0313) than Scramble (Figure 1G).

**Figure 2:**
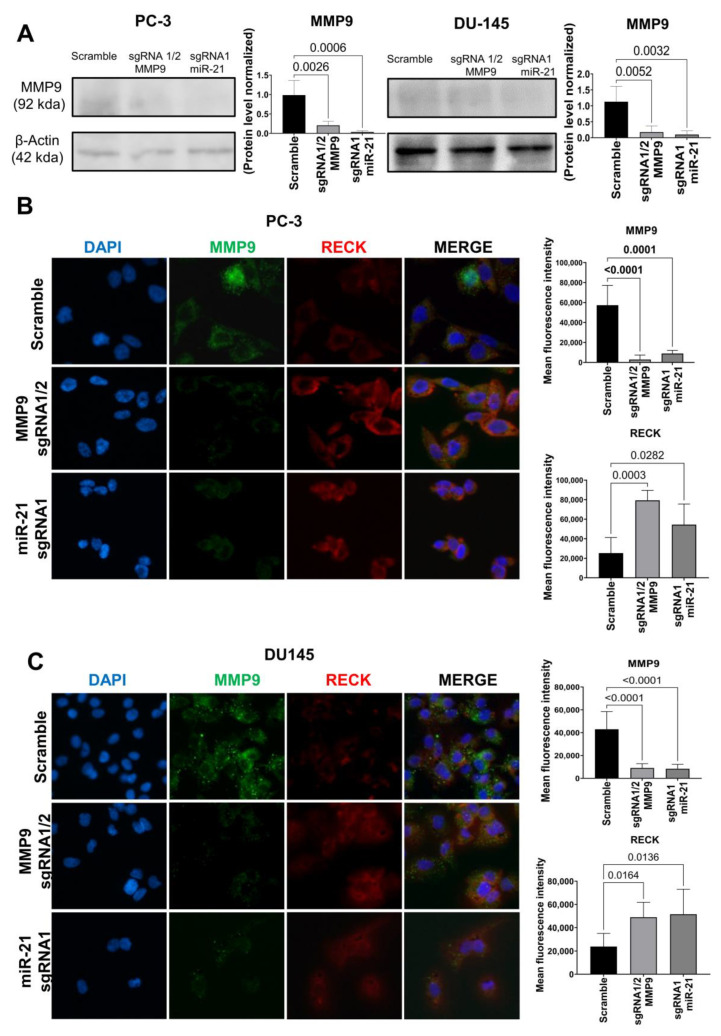
MMP9 and RECK protein expression in MMP9 and miR-21 CRISPR-Cas9-edited metastatic PCa cell lines. (**A**) Western blot analysis of MMP9 protein content in PC-3 and DU145 cells edited with MMP9 sgRNA 1/2 or miR-21 sgRNA1. (**B**,**C**) Protein immunofluorescence of colocalized MMP9 (green) and RECK (red) in samples edited with MMP9 sgRNA 1/2 or miR-21 sgRNA1 in both PC-3 and DU145 cells.

Next, we evaluated MMP9 protein expression using Western blot analysis to confirm the previous results. As shown in Figure 2A, MMP9 expression was downregulated in PC-3 cells, following CRISPR/Cas9 gene editing with MMP9 sgRNAs 1/2 (*p* = 0.0026) or miR-21 sgRNA1 (*p* = 0.0006), compared to Scramble. The same result was observed in DU145 cells, and gene editing with MMP9 sgRNAs 1/2 (*p* = 0.0052) or miR-21 sgRNA1 (*p* = 0.0032), compared to Scramble. These data were confirmed using immunofluorescence. MMP9 expression was downregulated in PC-3 cells, following CRISPR/Cas9 gene editing with MMP9 sgRNAs 1/2 (*p* < 0.0001) or miR-21 sgRNA1 (*p* < 0.0001), compared to Scramble. DU145 cells presented the same downregulation when gene editing with MMP9 sgRNAs 1/2 (*p* < 0.0001) or miR-21 sgRNA1 (*p* < 0.0001), compared to Scramble. Moreover, RECK protein expression was increased in PC-3 cells edited with MMP9 sgRNAs 1/2 (*p* = 0.003) or miR-21 sgRNA1 (*p* = 0.0282) compared to Scramble. The overexpression of RECK was also observed in DU145 cells when gene editing with MMP9 sgRNAs 1/2 (*p* = 0.0164) or miR-21 sgRNA1 (*p* = 0.0136), compared to Scramble (Figure 2B,C). No significative results were found in cells edited with miR-21 sgRNA 2 and MMP9 sgRNA 2 (Supplementary Figure S4). 

**Figure 3:**
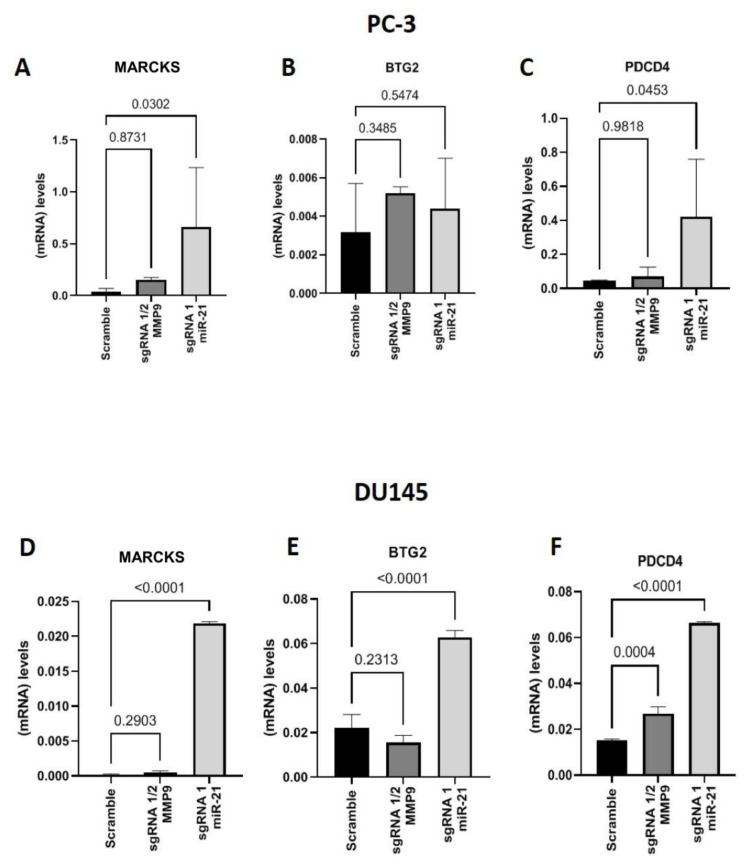
Gene expression of miR-21 targets in metastatic PCa cell lines. Gene expression of (**A**) MARKS, (**B**) BTG2, and (**C**) PDCD4 in MMP9 and miR-21 CRISPR-Cas9-edited PC-3 cells, and (**D**) MARKS, (**E**) BTG2, and (**F**) PDCD4 in MMP9 and miR-21 CRISPR-Cas9-edited DU145 cells.

After confirming the downregulation of miR-21 expression in the CRISPR/Cas9-edited PCa cell line, the gene expression of other miR-21 targets was assessed in the PC-3 and DU145 cells. The cells edited with miR-21 sgRNA1 displayed greater MARCKS expression (*p* = 0.0302) than Scramble (Figure 3A). On average, the mean BTG2 gene expression values in PC-3 cells with miR-21 sgRNA1 gene editing were greater than Scramble cells, but the results were not significantly different (Figure 3B). Additionally, the expression of apoptosis programmer PDCD4 was upregulated in miR-21 sgRNA1-edited samples compared to Scramble (*p* = 0.0453) (Figure 3C). Samples edited with MMP9 sgRNAs 1/2 did not present any significant differences compared to Scramble cells (Figure 3A–C).

In the DU145 cell line, miR-21 sgRNA1-edited cells displayed upregulated MARCKS (*p* < 0.0001) (Figure 3D), BTG2 (*p* < 0.0001) and PDCD4 (*p* < 0.0001) gene expression compared to Scramble cells (Figure 3D–F). On the other hand, MMP9 sgRNA 1/2-edited cells unaltered MARCKS and BTG2 (Figure 3E) expression, and upregulated PDCD4 (*p* < 0.0001) gene expression levels were observed compared to the Scramble control (Figure 3D–F).

**Figure 4:**
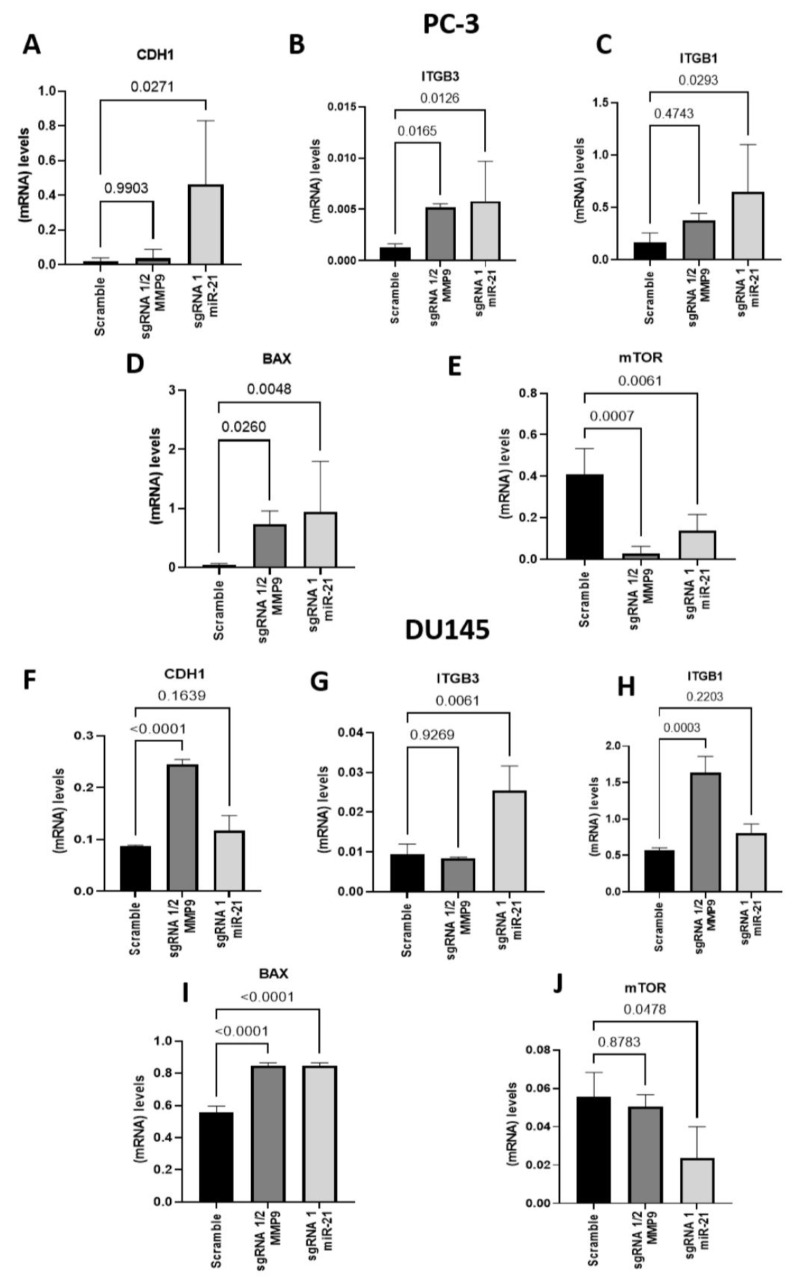
Gene expression of CDH1, integrins, BAX, and mTOR in metastatic PCa lines. Gene expression of (**A**) CDH1 cadherin, integrins (**B**) ITGB3 and (**C**) ITGB1, (**D**) BAX and (**E**) mTOR in MMP9 sgRNA 1/2- and miR-21 sgRNA1 CRISPR-Cas9-edited PC-3 cells. Gene expression of (**F**) CDH1 cadherin, integrins (**G**) ITGB3 and (**H**) ITGB1, (**I**) BAX and (**J**) mTOR in MMP9 sgRNA 1/2- and miR-21 sgRNA1 CRISPR-Cas9-edited DU145 cell line.

We also evaluated the gene expression of CDH1, ITGB3, and ITGB1, which MMPs degrade in the PC-3 and DU145 cell lines. In cells edited with miR-21 sgRNA1, CDH1, ITGB3 and ITGB1 expression levels were upregulated compared to Scramble (Figure 4A; *p* = 0.0271; Figure 4B; *p* = 0.0126; Figure 4C; *p* = 0.0293). Furthermore, on the other hand, ITGB1 and CDH1 gene expression were not altered in MMP9 sgRNA1/2-edited cells but ITGB3 was upregulated compared to Scramble (*p* = 0.0165) (Figure 4B). We also evaluated the gene expression of apoptosis and proliferation markers, BAX and mTOR, and found an increased expression of BAX in MMP9 sgRNA1/2 and miR-21 sgRNA1-edited cells compared to Scramble (MMP9 sgRNA 1/2, *p* = 0.0260; miR-21 sgRNA1, *p* = 0.0048) (Figure 4D). Concerning mTOR, the expression of this gene was downregulated in cells edited with MMP9 sgRNA 1/2 and miR-21 sgRNA1 when compared to Scramble (MMP9 sgRNA 1/2, *p* = 0.0007; miR-21 sgRNA1, *p* = 0.0061) (Figure 4E). 

In MMP9 sgRNA 1/2-edited DU145 cells, CDH1 gene expression was upregulated compared to the Scramble group (*p* < 0.0001), but there was no difference observed in cells edited with miR-21 sgRNA1 (Figure 4F). ITGB3 gene expression was higher in miR-21 sgRNA1-edited cells than Scramble cells (*p* = 0.0061) (Figure 4G). Moreover, ITGB1 gene expression in MMP9 sgRNA 1/2-edited cells was higher compared to Scramble (*p* = 0.0003) (Figure 4H). It was also determined that BAX gene expression was higher in cells edited with MMP9 sgRNA 1/2 and miR-21 sgRNA1 than Scramble (MMP9 sgRNA 1/2, *p* < 0.0001; miR-21 sgRNA1, *p* < 0.0001) (Figure 4I). Concerning mTOR gene expression, it was decreased in cells edited with miR-21 sgRNA1 (*p* = 0.0478) and unchanged in cells edited with MMP9 sgRNA 1/2 compared to the Scramble group (Figure 4J).

**Figure 5:**
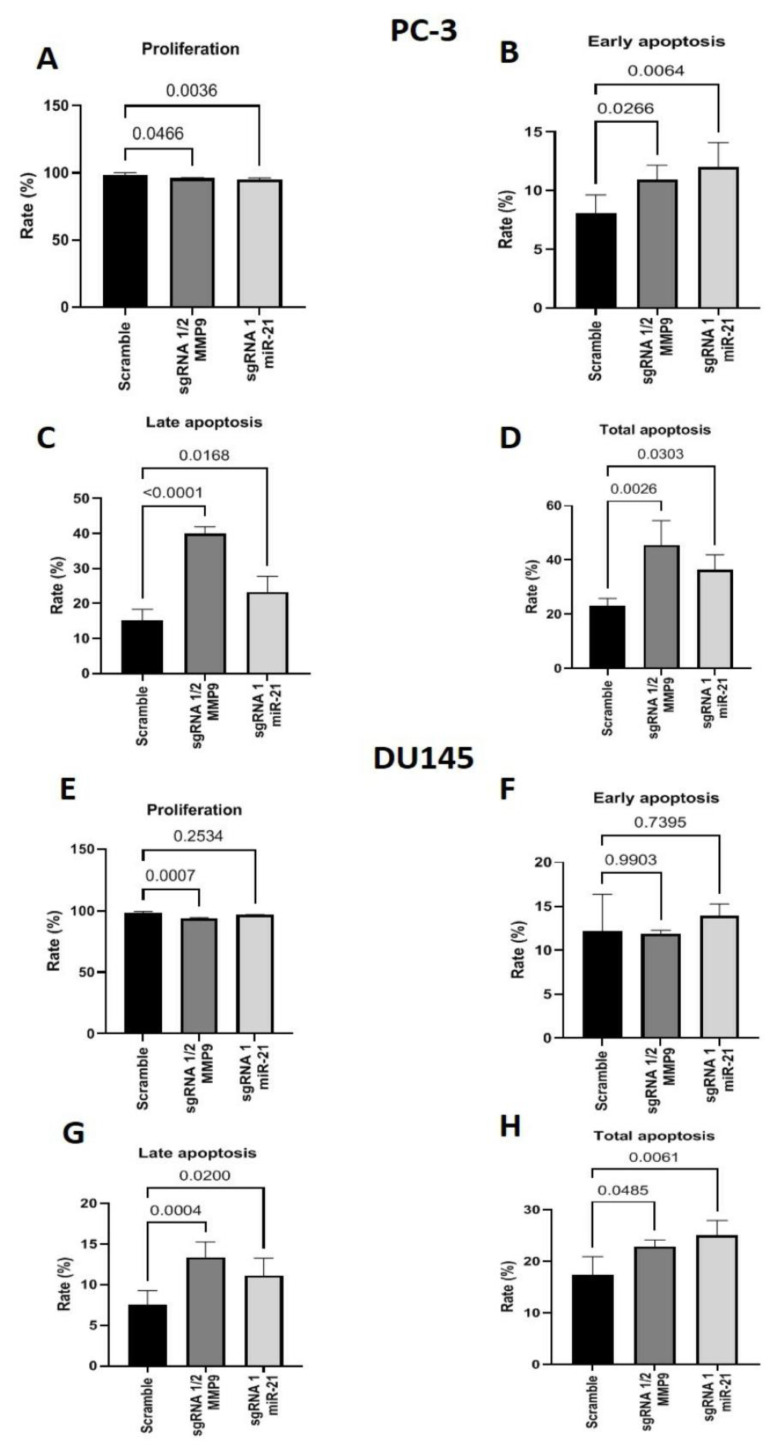
Flow cytometry for assessing proliferation and apoptosis in the metastatic PC-3 and DU145 cell lines. (**A**) Labeling MMP9 sgRNA1/2- and miR-21 sgRNA1 CRISPR-Cas9-edited PC-3 cells with a ki67 antibody to evaluate cell proliferation rate. (**B**–**D**) Labeling MMP9 sgRNA1/2 and miR-21 sgRNA1 CRISPR-Cas9-edited PC-3 cells with annexin-5 and 7-AAD to calculate the percentage of cells in the early and late stages of apoptosis and total apoptosis. (**E**) Labeling MMP9 sgRNA1/2 and miR-21 sgRNA1 CRISPR-Cas9-edited DU145 cells with a ki67 antibody to evaluate cell proliferation rate. (**F**–**H**) Labeling MMP9 sgRNA1/2 and miR-21 sgRNA1 CRISPR-Cas9-edited DU145 cells with annexin-5 and 7-AAD to calculate the percentage of cells in the early and late stages of apoptosis and total apoptosis.

Next, flow cytometry was performed with PC-3 (Figure 5A–D) and DU145 (Figure 5E–H) cells edited with MMP9 sgRNA 1/2 and miR-21 sgRNA1 to assess cell proliferation and apoptosis. In PC-3 cells, the proliferation rates of cells edited with MMP9 sgRNA 1/2 (*p* = 0.0456) or miR-21 sgRNA1 (*p* = 0.0036) were significantly attenuated compared to the Scramble group (Figure 5A). Additionally, the apoptosis assay revealed that MMP9 sgRNA 1/2-edited PC-3 cells display a higher rate of early (Figure 5B; *p* = 0.0266), late (Figure 5C; *p* < 0.0001), and total (Figure 5D; *p* = 0.0026) apoptosis than Scramble cells. A similar effect was also observed in miR-21 sgRNA1-edited PC-3 cells, which exhibited increased early (*p* = 0.0064) late (0.0168) and total (*p* = 0.0303) apoptosis compared to the Scramble group (Figure 5B–D, respectively). 

In the DU145 cell line, MMP9 sgRNA 1/2-edited cells displayed a decreased proliferation rate (*p* = 0.0007) compared to Scramble cells (Figure 5E). In contrast, the proliferation rate was unchanged in miR-21 sgRNA1-edited cells (Figure 5E). Neither MMP9 sgRNA 1/2 nor miR-21 gene editing affected the early apoptosis rate. However, both edited cell lines displayed increased late (MMP9 sgRNA 1/2 *p* = 0.0004; miR-21 sgRNA1, *p* = 0.02) and total (MMP9 sgRNA 1/2, *p* = 0.0485; miR-21 sgRNA1, *p* = 0.0061) apoptosis rates compared to the Scramble group (Figure 5F–H).

**Figure 6:**
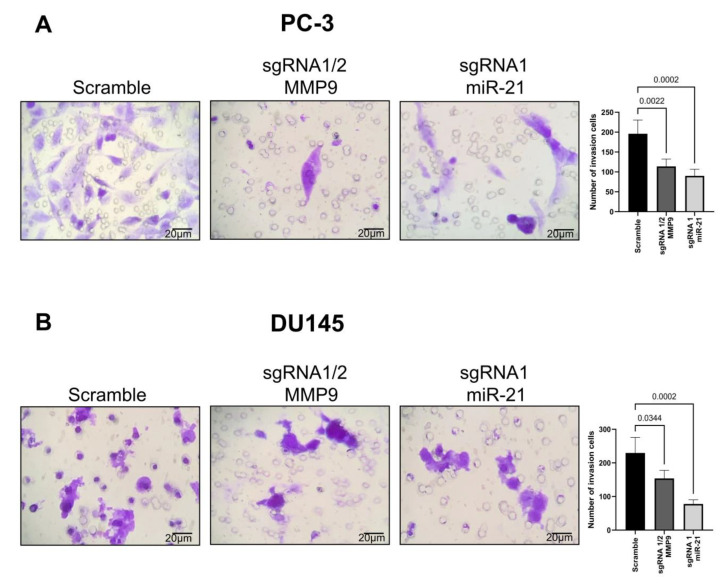
Transwell chamber invasion assay with metastatic (**A**) PC-3 and (**B**) DU145 CRISPR-Cas9 MMP9 sgRNA1/2- and miR-21 sgRNA1-edited cell lines.

Lastly, an invasion assay was conducted using MMP9 sgRNA1/2 and miR-21 sgRNA1 CRISPR-Cas9-edited PC-3 and DU145 cells to evaluate cell invasion potential. The invasion assay revealed that MMP9 sgRNA1/2 and miR-21 sgRNA1 CRISPR-Cas9-edited PC-3 cells contain fewer invasion cells (MMP9 sgRNA1/2, *p* = 0.0022; miR-21 sgRNA1, *p* = 0.0002) compared to the Scramble group (Figure 6A). Similarly, the number of invasion cells in DU145 cells edited with MMP9 sgRNA1/2 and miR-21 sgRNA1 was also less than observed in Scramble cells (MMP9 sgRNA1/2, *p* = 0.0344; miR-21 sgRNA1, *p* = 0.0002) (Figure 6B).

## 3. Discussion

CRISPR-Cas9 is a novel gene-editing technique that has brought several possibilities for gene therapies and corrections in multiple mutation-related diseases [18]. It has been proposed that this technique could potentially correct molecular mutations described in the literature or even downregulate or edit genes that may be possible candidates in molecular target therapy [19,20,21].

In this study, we sought to evaluate the role of MMP9 and the indirect regulator, miRNA-21, in metastatic PCa cell lines (PC-3 and DU145) using the CRISPR-Cas9 technique. We first standardized all the initial experiments by inserting the sgRNAs into the px-330 plasmid and expanding these samples in bacteria to generate a sufficient quantity of sgRNA-containing plasmids.

The sgRNA-containing plasmids were transfected into the PC-3 and DU-145 cell lines following the protocol of Zhang [22]. We opted to co-transfect two MMP9 sgRNAs to edit two different regions of MMP9 Exon 1, because a previous study showed that gene editing in more than one region could be more efficient [23]. Our approach resulted in a significant reduction in MMP9 gene and protein expression in PC-3 and DU-145 cells when using sgRNAs 1 and 2 for MMP9 in the same cell rather than with the individual sgRNAs. 

Furthermore, we also showed that miR-21 gene editing significantly decreased the expression of this gene in both PC-3 and DU145 cell lines. In both cell lines, miR-21-targeted downregulation was accompanied by the upregulation of target genes such as RECK, MARCKS, and PDCD4. It should be pointed out that the expression of the target gene BTG2 was increased in the DU145 cell line. These results are particularly interesting given that miR-21 is reportedly upregulated in patients with high-grade PCa [24].

The miR-21 target genes play important roles in key molecular mechanisms like cell migration and apoptosis. Indeed, Kim et al. (2020) demonstrated that miR-21 inhibition improved the stability and therapeutic efficacy and reduced metastasis in PCa xenografts in mice [25], a result consistent with our data. It is plausible that the increased expression of these key factors in miR-21 CRISPR-Cas9-edited cells could lead to reduced cell migration rates and higher cell apoptosis. We also observed upregulated BAX and PDCD4 gene and protein expression, two important apoptosis-related factors. In the PC-3 cell line, proliferation was decreased, and there were higher percentages of cells in early, late, and total apoptosis. In contrast, cell proliferation was unaffected in the DU145 cell line, but there were significant increases in apoptosis rates.

The results with MMP9 gene editing using CRISPR-Cas9 were similar to miR-21 editing. For example, MMP9 edited cells displayed altered gene and protein expression, reduced proliferation and increased apoptosis compared to the Scramble group. Until now, downregulated MMP9 gene and protein expression have not been linked to apoptosis. Thus, studies to investigate this cellular response were developed.

These experiments showed that MMP9-edited PC-3 and DU145 cells exhibit upregulated integrin gene expressions, including ITGB3 in PC-3 and CDH1 and ITGB1 in DU145 cells. The same response was observed in miR-21-edited cells, where CDH1, ITGB3, and ITGB1 were upregulated in PC-3 cells, and ITGB3 and ITGB1 were upregulated in DU145 cells. These results are particularly interesting because the relevance and importance of integrins in cancer progression are unclear.

A previous study found that the loss of CDH1 expression induces oncogenic cell transformation and facilitates tumor development [26]. Additionally, Werb et al. (2007) showed that metalloproteinases are responsible for cleaving the extracellular matrix and proteins such as CDH1 and other integrins [27]. Furthermore, Kurozumi et al. (2016) demonstrated that miRNA-223, which targets ITGA3 and ITGB1, acts as a tumor suppressor inhibiting these integrins [28]. Our data indicate that MMP9 downregulation may lead to upregulated integrin expression in PC-3 and DU145 cell lines, consequently reducing cell proliferation and stimulating apoptosis.

We evaluated the cell-invasion ability in gene-edited PC-3 and DU145 cell lines. The MMP9 and miR-21 edited cells contained a reduced number of invasion cells. This result is in line with Oh et al. (2014), who demonstrated that MMPs directly contribute to tumor invasion and metastasis by breaking down connective tissue barriers. Additionally, Bonci D et al. (2016) reported that the upregulation of miR-21 overtly activates TGF, facilitating tissue invasion and distant colonization, as occurs when prostate tumor cells develop metastases in bone tissue [3,29]. In this sense, our study results implicate MMP9 and miR-21 in metastatic PCa. Furthermore, we demonstrated that knocking these molecules out of metastatic cell lines using the CRISPR-Cas9 system slows cell proliferation and inhibits cell invasion, thus, highlighting the utility of this emerging technique for hard-to-treat diseases and conditions. Future studies in animal models are necessary to corroborate these in vitro findings.

The present study successfully downregulated the MMP9 and miR-21 gene and protein expression PCa cell lines using the CRISPR-Cas9 system. CRISPR-Cas9-edited PC-3 and DU145 cells displayed reduced cell proliferation, increased apoptosis and inhibited cell invasion, responses that could potentially trigger a regression in the metastatic PCa evolution.

## 4. Materials and Methods

### 4.1. Cell Culture

The present study utilized two metastatic PCa cell lines from the American Type Culture Collection (ATCC): PC-3 and DU145. These cells were cultured in MEM medium supplemented with 10% fetal bovine serum (FBS) and 1% antibiotic and antimycotic solution (Sigma Co., St. Louis, MO, USA). The cultures were incubated at 37 °C with 5% CO_2_, and the culture medium was changed every two days. The PC-3 and DU145 cell lines were certified (Supplementary Figure S1).

### 4.2. CRISPR-Cas9

The human MMP9 gene sequence (NM_004994.3) and miR-21 sequence (NR_029493.1) were used to design the single guide RNAs (sgRNA) for the knockout gene with the Optimized CRISPR Design tool (http://crispr.mit.edu/ (accessed on 5 May 2021)). We selected sgRNAs with high scores, which indicate that no off-target genomic sites were found. The selected MMP9 SgRNA sequences included CACCGgtgagaaccaatctcaccgac (Top1), AAACgtcggtgagattggttctcacC (Bottom1); and CACCGgaagggtggactggcgctgtc (Top2), AAACgacagcgccagtccacccttcC (Bottom2). Additionally, using the same methodology, we selected the following miR-21 sgRNAs: CACCggtcatggcaacaccagtcgat (Top1), AAACatcgactggtgttgccatgacC (Bottom1); and CACCggatgttgactgttgaatctca (Top2), AAACtgagattcaacagtcaacatcC (Bottom2). 

The phosphorylated sgRNAs were annealed and cloned into the px330-U6-GFP vector (Addgene, Watertown, MA, USA) using Addgene’s website instructions. The products of the cloning reactions were transformed into DH5α competent E. coli (Invitrogen, Waltham, MA USA). Individual bacterial colonies were selected and expanded, and the PureLink™ HiPure Plasmid Filter miniprep Kit (Invitrogen) was used to extract and isolate the plasmid. The constructs were sequenced with the following primers: FWD 5′-GGGCCTATTTCCCATGATTCC-3’ and REV 5’-CGCGCTAAA AACGGACTAGC-3’. Colonies harboring the px330-U6-GFP vector with sgRNA were again expanded, and the plasmids were extracted using the PureLink™ HiPure Plasmid Filter maxiprep Kit (Invitrogen). 

Next, these plasmids were transfected into the PC-3 and DU145 cell lines with the Xfect™ Transfection Reagent (Takara Bio, San Jose, CA, USA) following the manufacturer’s instructions. Both cells lines were exposed to 150 μg/mL puromycin dihydrochloride (Sigma Co.) for 10 days to select transfected cells. All the experiments were performed in three replicates of three independent experiments and standardized according to Zhang et al. [22].

### 4.3. RNA and MicroRNA Isolation and Real-Time PCR

The miRNA and mRNA were extracted from PC-3 and DU145 cells to evaluate gene expression levels with the mirVana kit (Ambion, Austin, TX, USA) following the manufacturer’s protocol. Sample concentration and purity were quantified spectrophotometrically by measuring the absorbance at 260 and 280 nm with a Nanodrop DN-1000 (Nanodrop, Wilmington, DE, USA). Samples were stored at −20 °C. 

Total RNA was converted into complementary DNA (cDNA) using the High Capacity cDNA Reverse Transcription Kit (Applied Biosystems, Foster City, CA, USA) at a concentration of 200 ng/µL. RT primers for miR-21 and RNU48 for the endogenous control, using TaqMan™ MicroRNA Reverse Transcription Kit, were performed according to the kit protocol (Applied Biosystems, CA, USA).

The qPCR reactions were performed in an ABI 7500 Fast thermal cycler (Applied Biosystems) operating in standard mode. Each reaction contained 2 µL of 5× HOT FIREPol^®^ Probe universal qPCR Mix (SOLIS BIODYNE, Estonia), 0.5 µL of the specific primer, 6.5 µL of nuclease-free water and 1.0 µL of cDNA. The following primers were used: MMP9 (Hs00957562_m1), RECK (Hs01019185_m1), MARCKS (Hs00158993_m1), BTG2 (Hs00198887_m1), PDCD4 (Hs00377253_m1), miR-21 (Hs04231424_s1), BAX (Hs00180269_m1), mTOR (Hs00234508_m1), CDH-1 (Hs01023895_m1), ITGB1 (Hs05351551_g1), ITGB3 (Hs01001469_m1) (Invitrogen). B2M was used for endogenous control. The Applied Biosystems Data Assist Software v3.01 was employed to analyze gene expression using the 2-ΔCT method. All reactions were performed in duplicate. The PCR cycling conditions were as follows: 2 min at 50 °C, 10 min at 95 °C; 40 cycles of 15 s at 95 °C and 1 min at 60 °C. 

### 4.4. Flow Cytometry for Cell Proliferation and Apoptosis

After CRISPR-Cas9 editing, the PC-3 and DU145 cells were evaluated with the Muse™ cell death kits (MCH100105) for Annexin V and a Proliferation kit (MCH100114) for Ki67, according to the manufacturer’s recommendations. The apoptosis and proliferation analyses were performed on a Muse^®^ Cell Analyzer (Merck Millipore, Burlington, MA, USA).

### 4.5. Western Blotting

Protein analysis was conducted using the total PC-3 and DU145 cell extracts. The samples were macerated in RIPA lysis buffer (Millipore, Billerica, MA, USA) with a TissueLyser (Qiagen, Germantown, MD, USA) and incubated at 4 °C for 20 min. These samples were then centrifuged at 15,000 rpm at 4 °C for 30 min.

The resulting supernatant was collected and mixed with 2× Laemmli sample buffer (Bio-Rad Laboratories, Hercules, CA, USA). Total protein samples (100 ng/mL) from both cell lines were loaded onto 10% acrylamide gels and subjected to SDS-PAGE. The separated proteins were then transferred to 0.45 µm pore-size nitrocellulose membranes at 120 V for 1 h. The SNAP apparatus (Millipore) was used for antibody incubation. Membranes were blocked with 3% bovine serum albumin (BSA, Sigma) diluted in Tris-buffered saline-Tween 20 (TBS-T) for 15 min. As suggested by the manufacturer, the primary antibodies were diluted in TBS-T with 1% BSA. The monoclonal MMP9 antibody utilized herein was applied at 1:1000 (Cloud—Clone). β-actin (1:1000, Millipore) was used to normalize the protein loading.

The membranes were incubated for 20 min at room temperature, washed with TBS-T 3 times for 15 s, and incubated with an anti-mouse IgG (H + L) secondary antibody, Human Serum Adsorbed, and Peroxidase labeled (KPL). Blots were developed using an ECL Western blotting detection system (Millipore), and the immunoblot images were captured with an Alliance 4.7 device (Uvitec, Cambridge, UK). Quantitative analyses were performed using the Alliance 16.06 software.

### 4.6. Immunofluorescence

Cells were fixed in 4% paraformaldehyde in PBS for 10 min at room temperature, washed 3 times with PBS and subjected to blocking solution with 3% BSA in PBS for 1 h. The MMP9 (1:150, Boster M00139) and RECK (1:100, Santa Cruz SC373929) antibodies were incubated in PBS containing 1% BSA overnight at 4 °C. For experiments assessing MMP9 expression, after washing, cells were incubated for 1 h at room temperature with Alexa Fluor 488 AffiniPure goat anti-rabbit (Jackson ImmunoResearch 111-545-003, West Grove, PA, USA) at 1:200. RECK determination was achieved using Alexa Fluor 647 AffiniPure goat anti-mouse (Jackson ImmunoResearch 115-605-003) at 1:200. The cells were washed and marked with ProLong™ Gold Antifade Mountant for nuclei staining with DAPI (Invitrogen). Cells were imaged on a Leica TCS SP2 II laser scanning microscope.

### 4.7. Invasion Assays

PC-3 and DU145 cell lines edited with CRISPR-Cas9 for MMP9 and miR-21, as well as their respective controls, were plated [approximately 12,000 cells in 250 µL of MEM serum-free culture medium (Gibco^TM^)] in Transwell chambers (Becton–Dickinson, São Paulo, SP, Brazil) with an 8 μm pore size containing 50 µL of Matrigel diluted in MEM serum-free culture medium (1:5). Next, 750 µL of culture medium containing 10% FBS was added to the lower chamber of the plate. The cells were maintained in a CO_2_ incubator for 48 h at 37 °C. Cells were fixed with 4% formaldehyde in PBS, stained with a 1% crystal violet solution in methanol, and counted on an optical microscope at 20× magnification.

### 4.8. Statistical Analysis 

A Kolmogorov–Smirnov normality test was performed for the expression and correlation analyses. A t-test was used when comparing two groups, and ANOVA was used for comparing three groups. The GraphPad Prism 9.0 software was used for all statistical analyses. Statistical significance was set at *p* < 0.05.

## Data Availability

The datasets used and/or analyzed during the current study are available to the corresponding author on reasonable request.

## Supplementary Materials

The following supporting information can be downloaded at: https://www.mdpi.com/article/10.3390/ijms241914847/s1.

## References

[1] Siegel R.L., Miller K.D., Fuchs H.E., Jemal A. (2021). Cancer Statistics, 2021. CA Cancer J. Clin..

[2] Lavaud P., Dumont C., Thibault C., Albiges L., Baciarello G., Colomba E., Flippot R., Fuerea A., Loriot Y., Fizazi K. (2020). Next-generation androgen receptor inhibitors in non-metastatic castration-resistant prostate cancer. Ther. Adv. Med. Oncol..

[3] Gong Y., Chippada-Venkata U.D., Oh W.K. (2014). Roles of matrix metalloproteinases and their natural inhibitors in prostate cancer progression. Cancers.

[4] Barillari G. (2020). The Impact of Matrix Metalloproteinase-9 on the Sequential Steps of the Metastatic Process. Int. J. Mol. Sci..

[5] Yuan J., Li W., Zhu J., Deng S., Tao X. (2020). Low expression of RECK in oral squamous cell carcinoma patients induces a shorter survival rate through an imbalance of RECK/MMPs. Int. J. Clin. Exp. Pathol..

[6] Reis S.T., Pontes-Junior J., Antunes A.A., de Sousa-Canavez J.M., Dall’Oglio M.F., Passerotti C.C., Abe D.K., Crippa A., Da Cruz J.A.S., Timoszczuk L.M. (2011). MMP-9 overexpression due to TIMP-1 and RECK underexpression is associated with prognosis in prostate cancer. Int. J. Biol. Markers.

[7] Ding L., Wang R., Shen D., Cheng S., Wang H., Lu Z., Zheng Q., Wang L., Xia L., Li G. (2021). Role of noncoding RNA in drug resistance of prostate cancer. Cell Death Dis..

[8] Ratti M., Lampis A., Ghidini M., Salati M., Mirchev M.B., Valeri N., Hahne J.C. (2020). MicroRNAs (miRNAs) and Long Non-Coding RNAs (lncRNAs) as New Tools for Cancer Therapy: First Steps from Bench to Bedside. Target. Oncol..

[9] McDonald A.C., Vira M., Walter V., Shen J., Raman J.D., Sanda M.G., Patil D., Taioli E. (2019). Circulating microRNAs in plasma among men with low-grade and high-grade prostate cancer at prostate biopsy. Prostate.

[10] Zhao W., Ning L., Wang L., Ouyang T., Qi L., Yang R., Wu Y. (2021). miR-21 inhibition reverses doxorubicin-resistance and inhibits PC3 human prostate cancer cells proliferation. Andrologia.

[11] Coppola V., Musumeci M., Patrizii M., Cannistraci A., Addario A., Maugeri-Saccà M., Biffoni M., Francescangeli F., Cordenonsi M., Piccolo S. (2013). BTG2 loss and miR-21 upregulation contribute to prostate cell transformation by inducing luminal markers expression and epithelial-mesenchymal transition. Oncogene.

[12] Li T., Li D., Sha J., Sun P., Huang Y. (2009). MicroRNA-21 directly targets MARCKS and promotes apoptosis resistance and invasion in prostate cancer cells. Biochem. Biophys. Res. Commun..

[13] Leite K.R., Reis S.T., Viana N., Morais D.R., Moura C.M., Silva I.A., Katz B., Srougi M. (2015). Controlling RECK miR21 Promotes Tumor Cell Invasion and Is Related to Biochemical Recurrence in Prostate Cancer. J. Cancer.

[14] Lentsch E., Li L., Pfeffer S., Ekici A.B., Taher L., Pilarsky C., Grützmann R. (2019). CRISPR/Cas9-Mediated Knock-Out of Kras. Int. J. Mol. Sci..

[15] Bodey B., Siegel S.E., Kaiser H.E. (2001). Immunocytochemical detection of matrix metalloproteinase expression in prostate cancer. In Vivo.

[16] Babichenko I.I., Andriukhin M.I., Pulbere S., Loktev A. (2014). Immunohistochemical expression of matrix metalloproteinase-9 and inhibitor of matrix metalloproteinase-1 in prostate adenocarcinoma. Int. J. Clin. Exp. Pathol..

[17] Folini M., Gandellini P., Longoni N., Profumo V., Callari M., Pennati M., Colecchia M., Supino R., Veneroni S., Salvioni R. (2010). miR-21: An oncomir on strike in prostate cancer. Mol. Cancer.

[18] Liu T., Shen J.K., Li Z., Choy E., Hornicek F.J., Duan Z. (2016). Development and potential applications of CRISPR-Cas9 genome editing technology in sarcoma. Cancer Lett..

[19] Fei T., Chen Y., Xiao T., Li W., Cato L., Zhang P., Cotter M.B., Bowden M., Lis R.T., Zhao S.G. (2017). Genome-wide CRISPR screen identifies HNRNPL as a prostate cancer dependency regulating RNA splicing. Proc. Natl. Acad. Sci. USA.

[20] Wei C., Wang F., Liu W., Zhao W., Yang Y., Li K., Xiao L., Shen J. (2018). CRISPR/Cas9 targeting of the androgen receptor suppresses the growth of LNCaP human prostate cancer cells. Mol. Med. Rep..

[21] Ye R., Pi M., Cox J.V., Nishimoto S.K., Quarles L.D. (2017). CRISPR/Cas9 targeting of GPRC6A suppresses prostate cancer tumorigenesis in a human xenograft model. J. Exp. Clin. Cancer Res..

[22] Ran F.A., Hsu P.D., Wright J., Agarwala V., Scott D.A., Zhang F. (2013). Genome engineering using the CRISPR-Cas9 system. Nat. Protoc..

[23] Ran F.A., Hsu P.D., Lin C.Y., Gootenberg J.S., Konermann S., Trevino A.E., Scott D.A., Inoue A., Matoba S., Zhang Y. (2013). Double nicking by RNA-guided CRISPR Cas9 for enhanced genome editing specificity. Cell.

[24] Arisan E.D., Rencuzogullari O., Freitas I.L., Radzali S., Keskin B., Kothari A., Warford A., Uysal-Onganer P. (2020). Upregulated Wnt-11 and miR-21 Expression Trigger Epithelial Mesenchymal Transition in Aggressive Prostate Cancer Cells. Biology.

[25] Kim K., Kim H.H., Lee C.H., Kim S., Cheon G.J., Kang K.W., Chung J.-K., Youn H. (2020). Therapeutic efficacy of modified anti-miR21 in metastatic prostate cancer. Biochem. Biophys. Res. Commun..

[26] Olson A., Le V., Aldahl J., Yu E.J., Hooker E., He Y., Lee D.-H., Kim W.K., Cardiff R.D., Geradts J. (2019). The comprehensive role of E-cadherin in maintaining prostatic epithelial integrity during oncogenic transformation and tumor progression. PLoS Genet..

[27] Page-McCaw A., Ewald A.J., Werb Z. (2007). Matrix metalloproteinases and the regulation of tissue remodelling. Nat. Rev. Mol. Cell Biol..

[28] Kurozumi A., Goto Y., Matsushita R., Fukumoto I., Kato M., Nishikawa R., Sakamoto S., Enokida H., Nakagawa M., Ichikawa T. (2016). Tumor-suppressive microRNA-223 inhibits cancer cell migration and invasion by targeting ITGA3/ITGB1 signaling in prostate cancer. Cancer Sci..

[29] Bonci D., Coppola V., Patrizii M., Addario A., Cannistraci A., Francescangeli F., Pecci R., Muto G., Collura D., Bedini R. (2016). A microRNA code for prostate cancer metastasis. Oncogene.

