# Supplementary material for: The Effect of Gene Editing by CRISPR-Cas9 of miR-21 and the Indirect Target MMP9 in Metastatic Prostate Cancer
Source: Int J Mol Sci. 2023 Oct 3;24(19):14847. doi: 10.3390/ijms241914847 (PMC10573678; doi:10.3390/ijms241914847)

Supplementary Figure S1. The PC-3 and DU145 cell lines were certified.

DTAPEP
BIOTÉRIO CENTRAL

DTAPEP
Diretoria Técnica de Apoio ao Ensino e Pesquisa

MEDICINA USP

AUTENTICAÇÃO DE LINHAGENS

Laudo do Perfil de STR´s / STR Profile Report

INFORMAÇÃO DO CLIENTE / *CUSTOMER INFORMATION*

**Nome** / *Name*	IRAN AMORIM DA SILVA
**Instituição** / *Institution*	HCFMUSP
**Departamento** / *Department*	CIRURGIA
**Telefone** / *Phone*	1130617183
**E-mail**	iransilva@gmail.com

**Nome da Linhagem** / *Cell line name*	PC3 (ATCC CRL-1435)

**Amostra recebida em** / *Samples received in* 00-00-0000	**Data do laudo** / *Report date*: 16-12-2019

Loci	**Resultado do Teste** / *Test Result* (**amostra**/*sample*)				**Perfil da referência no Banco de Dados** / *Reference Database Profile*				
	**Perfil da Amostra** / *Query Profile* PC3				**Perfil da referência** / *Database Profile* PC3 (ATCC CRL-1435)				
**TH01**	6	7			6	7			
**TPOX**	8	9			8	9			
**vWA**	17				17				
**CSF1PO**	11				11				
**D16S539**	11				11				
**D7S820**	8	11			8	11			
**D13S317**	11				11				
**D5S818**	13				13				
**D21S11**									
**Amelogenin**	x				x				
**Número de alelos iguais entre a amostra e referência** / *Number of shared alleles between query sample and database profile*:									12
**Total de alelos na amostra referência** / *Total number of alleles in the database profile*:									12
**Porcentagem de correspondência entre alelos da amostra e da referência** / *Percent match between the submitted sample and the database profile*:									100

RESULTADO / *RESULT*
Linhagens celulares com uma porcentagem de correspondência >=80% são consideradas relacionadas, ou seja, derivada de um ancestral comum. Linhagens com porcentagem de correspondência entre 55% e 80% necessitam de novos testes (maior número de marcadores) para confirmar sua autenticidade. / *Cell lines with >=80% match are considered to be related; i.e., derived from a common ancestry. Cell lines with between a 55% to 80% match require further profiling for authentication of relatedness. (ATCC - STR Profile Report)*

METODOLOGIA / *METHODOLOGY*
10 STR's, correspondendo aos loci TH01, TPOX, vWA, CSF1PO, D16S539, D7S820, D13S317, D5S818, Amelogenin e D21S11 (GenePrint 10 / Promega), foram amplificados e submetidos à eletroforese capilar para separação dos fragmentos (3730 DNA Analyzer - Applied Biosystems). Os dados foram analisados através do software XXX GeneMaker HDI v.1.1.0 (Softgenetics LCC). Controles positivos e negativos apropriados foram utilizados na validação de cada amostra. / *10 STR's (TH01, TPOX, vWA, CSF1PO, D16S539, D7S820, D13S317, D5S818, Amelogenin and D21S1- GenePrint 10 / Promega) are co-amplified and submitted to capillary electrophoresis (3730 DNA Analyzer - Applied Biosystems). An internal lane standard (ILS) and allelic ladder are provided for sizing and genotyping of amplified fragments, and the 2800M Control DNA is supplied as a positive control (GenePrint 10 / Promega). Data is analyzed using software GeneMaker HDI v.1.1.0 (Softgenetics LCC).*

INTERPRETAÇÃO DOS DADOS / *INTERPRETATION OF DATA*
Os resultados foram interpretados segundo as diretrizes do ANSI Standards 2011 (ASN-0002 / ATCC Standards Development Organization) / *Results were interpreted as described in ANSI Standards 2011 (ASN-0002 / ATCC Standards Development Organization / ATCC STR Profile Report)*

☐ **A linhagem enviada para análise é de origem humana, mas o seu perfil não corresponde a nenhuma linhagem padrão presente nos bancos de dados analisados** / *The submitted sample profile is of human origin, but not a match for any profile in the STR database*

X **A linhagem enviada para análise corresponde exatamente à seguinte linhagem celular humana** / *The submitted profile is an exact match for the following human cell lines):*
PC-3 Prostate Adenocarcinoma Human (ATCC:CRL-1435)

☐ **O perfil de marcadores da linhagem enviada é similar à(s) seguinte(s) linhagem(s) celular(es) humana(s)** / *The submitted profile is similar to the following ATCC human cell line(s):*

OBSERVAÇÕES / *Additional Comments*
https://www.atcc.org/en/STR_Database.aspx

Responsável Técnico: Christian Albert Merkel
Email: c.merkel@fm.usp.br

AUTENTICAÇÃO DE LINHAGENS

Laudo do Perfil de STR´s / STR Profile Report

INFORMAÇÃO DO CLIENTE / *CUSTOMER INFORMATION*

**Nome** / *Name*	IRAN AMORIM DA SILVA
**Instituição** / *Institution*	HCFMUSP
**Departamento** / *Department*	CIRURGIA
**Telefone** / *Phone*	1130617183
**E-mail**	iransilva@gmail.com

**Nome da Linhagem** / *Cell line name*	DU145 (ATCC HTB-81)

**Amostra recebida em** / *Samples received in* 00-00-0000	**Data do laudo** / *Report date*: 16-12-2019

Loci	**Resultado do Teste** / *Test Result* (**amostra**/*sample*)				**Perfil da referência no Banco de Dados** / *Reference Database Profile*				
	**Perfil da Amostra** / *Query Profile* DU145 (ATCC HTB-81)				**Perfil da referência** / *Database Profile* DU145 (ATCC HTB-81)				
**TH01**	7				7				
**TPOX**	10	11			11				
**vWA**	17	18			17	18	19		
**CSF1PO**	10	11			10	11			
**D16S539**	10	11	13		11	13			
**D7S820**	7	10	11		7	10	11		
**D13S317**	11	12	13	14	12	13	14		
**D5S818**	10	13			10	13			
**D21S11**									
**Amelogenin**	x	y			x	y			
**Número de alelos iguais entre a amostra e referência** / *Number of shared alleles between query sample and database profile*:									18
**Total de alelos na amostra referência** / *Total number of alleles in the database profile*:									20
**Porcentagem de correspondência entre alelos da amostra e da referência** / *Percent match between the submitted sample and the database profile*:									95

RESULTADO / *RESULT*
Linhagens celulares com uma porcentagem de correspondência >=80% são consideradas relacionadas, ou seja, derivada de um ancestral comum. Linhagens com porcentagem de correspondência entre 55% e 80% necessitam de novos testes (maior número de marcadores) para confirmar sua autenticidade. / *Cell lines with >=80% match are considered to be related; i.e., derived from a common ancestry. Cell lines with between a 55% to 80% match require further profiling for authentication of relatedness. (ATCC - STR Profile Report)*

METODOLOGIA / *METHODOLOGY*
10 STR's, correspondendo aos loci TH01, TPOX, vWA, CSF1PO, D16S539, D7S820, D13S317, D5S818, Amelogenin e D21S11 (GenePrint 10 / Promega), foram amplificados e submetidos à eletroforese capilar para separação dos fragmentos (3730 DNA Analyzer - Applied Biosystems). Os dados foram analisados através do software XXX GeneMaker HDI v.1.1.0 (Softgenetics LCC). Controles positivos e negativos apropriados foram utilizados na validação de cada amostra. / *10 STR's (TH01, TPOX, vWA, CSF1PO, D16S539, D7S820, D13S317, D5S818, Amelogenin and D21S1- GenePrint 10 / Promega) are co-amplified and submitted to capillary electrophoresis (3730 DNA Analyzer - Applied Biosystems). An internal lane standard (ILS) and allelic ladder are provided for sizing and genotyping of amplified fragments, and the 2800M Control DNA is supplied as a positive control (GenePrint 10 / Promega). Data is analyzed using software GeneMaker HDI v.1.1.0 (Softgenetics LCC).*

INTERPRETAÇÃO DOS DADOS / *INTERPRETATION OF DATA*
Os resultados foram interpretados segundo as diretrizes do ANSI Standards 2011 (ASN-0002 / ATCC Standards Development Organization) / *Results were interpreted as described in ANSI Standards 2011 (ASN-0002 / ATCC Standards Development Organization / ATCC STR Profile Report)*

☐ **A linhagem enviada para análise é de origem humana, mas o seu perfil não corresponde a nenhuma linhagem padrão presente nos bancos de dados analisados** / *The submitted sample profile is of human origin, but not a match for any profile in the STR database*

☐ **A linhagem enviada para análise corresponde exatamente à seguinte linhagem celular humana** / *The submitted profile is an exact match for the following human cell lines)*:

X **O perfil de marcadores da linhagem enviada é similar à(s) seguinte(s) linhagem(s) celular(es) humana(s)** / *The submitted profile is similar to the following ATCC human cell line(s)*:
DU 145 Prostate Carcinoma Human (ATCC HTB-81)

OBSERVAÇÕES / *Additional Comments*
https://www.atcc.org/en/STR_Database.aspx

Responsável Técnico: Christian Albert Merkel
Email: c.merkel@fm.usp.br

Supplementary Figure S2. Concentration and time for selecting plasmid-transfected cells with puromycin.

Control

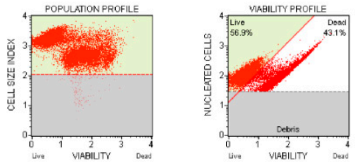

Viable Cells / mL :	1.49E+07
Viability % :	56.88 %
Total Cells / mL :	2.62E+07
Events Acquired :	20164
Dilution Factor :	20.00
Original Volume :	10.00 mL

100 µg/mL

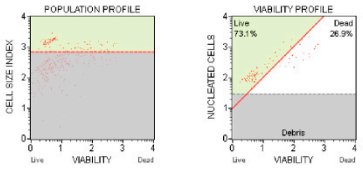

Viable Cells / mL :	1.60E+04
Viability % :	73.08 %
Total Cells / mL :	2.18E+04
Events Acquired :	286
Dilution Factor :	20.00
Original Volume :	10.00 mL

150 µg/mL

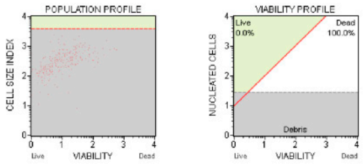

Viable Cells / mL :	0.00E+00
Viability % :	0.00 %
Total Cells / mL :	0.00E+00
Events Acquired :	266
Dilution Factor :	20.00
Original Volume :	10.00 mL

200 µg/mL

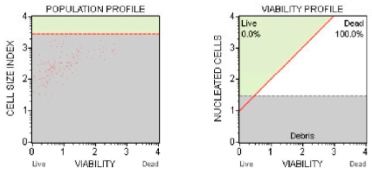

Viable Cells / mL :	0.00E+00
Viability % :	0.00 %
Total Cells / mL :	0.00E+00
Events Acquired :	189
Dilution Factor :	20.00
Original Volume :	10.00 mL

250 µg/mL

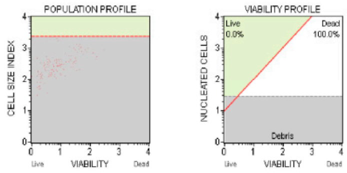

Viable Cells / mL :	0.00E+00
Viability % :	0.00 %
Total Cells / mL :	0.00E+00
Events Acquired :	137
Dilution Factor :	10.00
Original Volume :	10.00 mL

Supplementary Figure S3. Validation of plasmid transfection with GFP

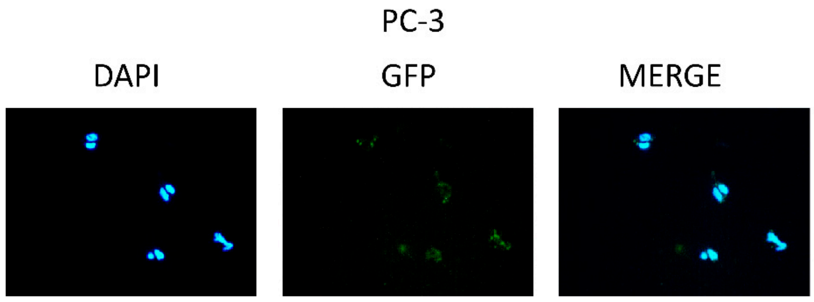

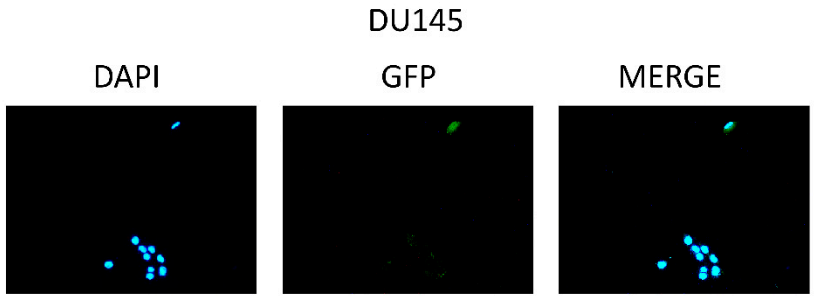

Supplementary Figure S4.

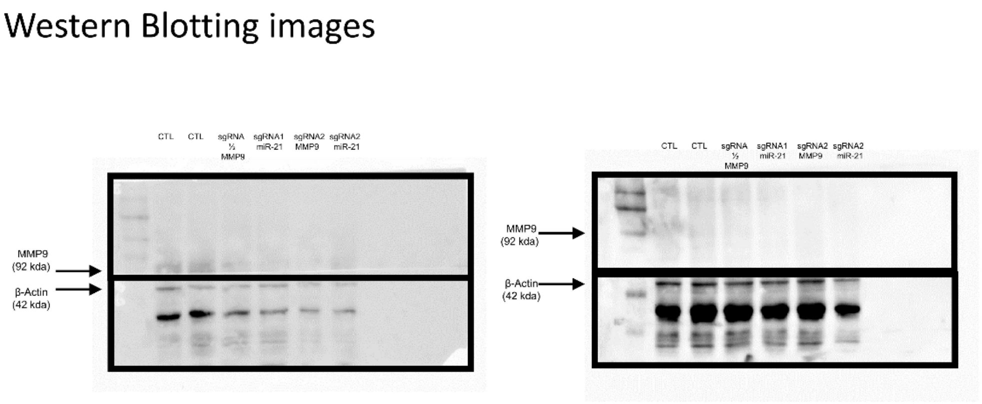

Supplement: Supplementary file 1 [file ijms-24-14847-s001.zip › ijms-2515546-supplementary.pdf]
